# Evaluation of the BD Phoenix CPO detect panel for prediction of Ambler class carbapenemases

**DOI:** 10.1038/s41598-021-92336-3

**Published:** 2021-06-23

**Authors:** Daniel Jonas, Sandra Reuter, Sarah Klassen, Sabine Weber, Marion Buck, Tommaso Giani, Gian Maria Rossolini, Hajo Grundmann

**Affiliations:** 1 Institute for Infection Prevention and Hospital Epidemiology, Faculty of Medicine, University of Freiburg, Freiburg, Germany; 2 Department of Experimental and Clinical Medicine, University of Florence, Florence, Italy; 3 Clinical Microbiology and Virology Unit, Florence Careggi University Hospital, Florence, Italy

**Keywords:** Antimicrobials, Bacteriology, Clinical microbiology, Infectious-disease diagnostics, Microbiology, Microbiology techniques, Infectious diseases

## Abstract

Rapid detection of carbapenemases as a cause of resistance is beneficial for infection control and antimicrobial therapy. The BD Phoenix NMIC-502 panel and CPO detect test identifies presence of carbapenemases in Enterobacterales such as *Klebsiella pneumoniae* and assigns them to Ambler classes. To evaluate the performance of the CPO detect panel, we employed a European collection of 1222 *K. pneumoniae* including carbapenem non-susceptible and susceptible clinical isolates from 26 countries, for which draft genomes were available after Illumina sequencing and the presence of carbapenemase genes had been identified by ARIBA gene calling. The CPO panel detected 488 out of 494 carbapenemase-encoding isolates as positive and six as negative. One-hundred and two isolates were tested positive for carbapenemase in the absence of any carbapenemase gene. The CPO panel identified 229 out of 230 KPC-positive isolates as carbapenemase producing and classified 62 of these as class A enzyme. Similarly, the CPO panel correctly specified 167 of 182 as class D. Regarding metallo-beta-lactamases, the CPO panel assigned 78 of 90 MBL positive isolates to class B enzymes. The sensitivity of the CPO panel in detecting carbapenemase activity was 99.5%, 97.7% and 98.3% for class A, B and D enzymes, respectively. The sensitivity in assignation to Ambler class A, B and D was 27%, 86% and 91%, respectively. An overall sensitivity of 98.8% and specificity of 86% in unclassified detection of carbapenemases was observed, with frequent false positive detection of carbapenemase producing organisms, thus rendering further confirmatory tests necessary.

## Introduction

There has been an increasing number of reports on the spread of carbapenem-resistant Gram-negative pathogens including Enterobacterales (CRE), Gram-negative non-fermenters such as *Pseudomonas aeruginosa* and *Acinetobacter* spp., as well as carbapenemase-producing organisms (CPO) and their limited treatment options^1–3^.

In principle, carbapenem resistance can be caused by (i) reduced cell permeability to antibiotics due to loss of influx porins or increased efflux pump activity, possibly in combination with the production of extended-spectrum β-lactamases (ESBL) or AmpC-type β-lactamases, or by (ii) production of inactivating enzymes, i.e., carbapenemases. While the former mechanisms arise from mutations and may impair biological fitness^4–6^, presumably being rather more self-limiting, carbapenemases in general are of major concern, especially since they are often encoded on mobile genetic elements, which can spread rapidly by lateral gene transfer across genetic and species boundaries. Distinguishing between these two principal resistance mechanisms can therefore be both meaningful and guiding from the point of view of infection control. Moreover, detailed classification of carbapenemases can be valuable for therapy, since the new beta-lactamase inhibitor combinations have only a spectrum limited to serine carbapenemases of class A (and in some cases to class D), while only monobactams are stable to class B metallo-beta-lactamases^7–9^.

For this reason, there is increasing interest in the detection of carbapenemase production in the diagnostic microbiology laboratory. Several diagnostic tests have been developed to prove production of carbapenemases, e.g. the Carba NP-direct test^10^, the modified carbapenem inactivation method (mCIM)^11^, or the modified Hodge test^12,13^. However, these tests do not differentiate between the carbapenemase class, which can be determined either by phenotypic tests using inhibitors^14,15^ or by tests that detect the enzymes by lateral-flow immunochromatography^16,17^, or the carbapenemase genes by molecular detection methods using PCR^18,19^, micro-arrays^20,21^ or whole genome sequencing (WGS)^22–24^. However, all these tests must be performed in addition to conventional antimicrobial susceptibility testing (AST), and are usually carried out with carbapenem-resistant isolates after the results of AST are available, leading to some delay in the information released.

The BD Phoenix is a bacterial growth-based automated system for determination of antibiotic resistance characteristics by use of various test panels. The “CPO detect test” is embedded in the NMIC-502 panel of the BD Phoenix card for AST of Gram-negative pathogens (in short „the CPO panel”), which provides a means to (i) detect and (ii) classify carbapenemases in line with AST, and without any further workload, utilizing bacterial growth in the presence of meropenem, doripenem, temocillin and cloxacillin, both alone and in combination with various chelators and beta-lactamase inhibitors (Becton Dickinson, instructions for use of the test).

Few previous studies have investigated the performance of the CPO detect test^25–30^. These studies are mainly based on smaller collections of locally or regionally collected/sampled CPO-positive strains and just some 30–50 CPO-negative control strains selected on the basis of resistance without carbapenemase activity—which has already been discussed as a limitation in assessment of test specificity.

The study presented here is a large evaluation of the performance of the CPO detection test included in the BD Phoenix^™^ NMIC-502 panel for (i) detection and (ii) classification of carbapenemases. It is based on a representative European collection of over 1000 *Klebsiella pneumoniae* isolates from 26 countries which originated from surveillance of carbapenem resistance throughout Europe in 2014, and all of which had previously been subjected to WGS analysis^31^. Of note, this collection not only included carbapenem-resistant isolates but also an equal number of non-resistant isolates from microbiological routine diagnostics throughout Europe.

## Results

In accordance with AST, the CPO panel can distinguish carbapenem resistances due to carbapenemase production from those mediated by other mechanisms (e. g. altered permeability) in Enterobacterales such as *Klebsiella pneumoniae*. The phenotypes of unspecified carbapenemases are designated CARB, whereas class A, B or D enzymes are flagged as CARBA, CARBB or CARBD. While the test only indicates one of the four possible results, the following analysis distinguishes between the detection of any carbapenemases (CARB_OR), regardless of class, and the more precise and specific assignment to one of the three classes.

For a set of 1222 *K. pneumoniae,* data on the presence/absence of carbapenemase resistance genes and multilocus sequence types were available for subsequent analyses based on draft genome sequencing.

Carbapenemases belonging to three Ambler classes, namely class A (including KPC-2, -3 and -12), class B metallo-beta-lactamases (MBL of IMP-1, NDM-1, VIM-1 and -4 type) and class D carbapenemases (including OXA-48-like [OXA 48, 162, 204, 232]) were identified by employing ARIBA gene identification by local assembly.

Of the 1222 isolates, 503 contained KPC, MBL and OXA carbapenemase genes, according to Illumina sequence data. Resolution of 15 initially discrepant results between positive sequence data and negative phenotypic CPO panel results using gene-specific PCR resulted in 9 PCR negative results and 6 strains that remained positive for carbapenemase sequences based on both the sequence data and the PCR results. In the following, 494 isolates were thus considered genotypically positive and 728 genotypically negative.

In 488 of 494 genotypic positive strains, the CPO panel detected carbapenemases irrespective of any of the four particular phenotypes (CARB, CARBA, CARBB or CARBD) (overall sensitivity 98.8%). None of the four CPO tests detected the presence of a carbapenemase phenotype in six of 494 isolates with a carbapenemase genotype (Table 1). According to sequence data, these strains with false negative CPO test result contained sequences coding for the following carbapenemases confirmed by PCR: OXA-48-like (3), KPC3 (1), VIM-1-like (2).

**Table 1:** CPO panel for (i) detection or (ii) classification of (un-)classified carbapenemase phenotypes (columns) in 1222 genotyped strains (rows).

Carbapenemase encoding genotype/Class—(CPO carbapenemase phenotype)	CPO detect	
	Negative	Positive
**Any carbapenemases encoding DNA—(CARB, CARBA, CARBB OR CARBD)**		
Neg.	626	102
Pos.	6	488
**Class A—(CARBA)**		
Neg.	977	15
Pos.	168	62
**Class B—(CARBB)**		
Neg.	1122	10
Pos.	12	78
**Class D—(CARBD)**		
Neg.	1038	2
Pos.	15	167
**Any carbapenemases encoding DNA (CARB)**		
Neg.	652	76
Pos.	314	180

Phenotypes have been placed in parentheses. The abundancy of phenotypical classifications (CARBA, CARBB and CARBD) in strains with genotypical classes A, B or D are detailed. For basic detection of carbapenemases regardless of their classification, CPO detect results for carbapenemase phenotypes without further details (CARB) or with any of the four possible data outputs (CARB, CARBA, CARBB or CARBD) are compared with the presence or absence of any carbapenemase encoding DNA, i.e., regardless of the Ambler classification of the gene.

Of 728 isolates without detectable carbapenemase encoding genes, the CPO test produced a negative result in 626 strains, with a specificity of 86.0%. One hundred and two isolates tested phenotypically positive for carbapenemase in the absence of identification of any known carbapenemase coding genes in the draft genome data, giving an accuracy of 91.2% and a negative or positive predictive value of 99.1% or 82.7%, respectively, for detection of carbapenemases. For a more discriminant analysis, especially of the discrepant test results, CPO detection results were considered in parallel with the MIC values measured for meropenem or temocillin (Table 2). According to the NMIC-502 panel AST, most (90) of these 102 (any CARB) phenotypically false positive isolates had—only a slightly/moderately increased MIC against meropenem of 0.25–8 mg/L, in contrast to 342 of 488 concordantly positive isolates with an MIC of > 8 mg/L (Table 2).

**Table 2:** MIC distribution and CPO panel detection of (un-)classified carbapenemase phenotypes (columns) in genotyped strains (rows).

Any carbape-nemase genotype	Any CPO carbapenemase (CARB, CARBA, CARBB or CARBD) detected																	
	None									Any								Sum
	Meropenem MIC (CPO tested)								Sum of none detected	Meropenem MIC (CPO tested)							Sum of any detected	
	≤ 0.125	0.25	0.5	1	2	4	8	> 8		0.25	0.5	1	2	4	8	> 8		
None	581	28	5	5	4	3			626	8	9	9	22	25	17	12	102	728
Any	2		1		1			2	6	5	19	17	45	41	19	342	488	494
Sum	583	28	6	5	5	3		2	632	13	28	26	67	66	36	354	590	1222

Any carbape-nemase genotype	CPO unspecified carbapenemase (CARB) detected																	
	CARB absent									CARB present								Sum
	Meropenem MIC (CPO tested)								Sum of CARB absent	Meropenem MIC (CPO tested)							Sum of CARB present	
	≤ 0.125	0.25	0.5	1	2	4	8	> 8		0.25	0.5	1	2	4	8	> 8		
None	581	35	10	10	7	5	1	3	652	1	4	4	19	23	16	9	76	728
Any	2	3	17	16	44	41	19	172	314	2	3	1	2			172	180	494
Sum	583	38	27	26	51	46	20	175	966	3	7	5	21	23	16	181	256	1222

Class A carbape-nemase genotype	CPO class A carbapenemase (CARBA) detected																	
	CARBA absent									CARBA present								Sum
	Meropenem MIC (CPO tested)								Sum of CARBA absent	Meropenem MIC (CPO tested)							Sum of CARBA present	
	≤ 0.125	0.25	0.5	1	2	4	8	> 8		0.25	0.5	1	2	4	8	> 8		
Neg.	583	34	31	29	69	67	32	132	977	6	3	2	2			2	15	992
Pos.		1			1			166	168					2	4	56	62	230
Sum	583	35	31	29	70	67	32	298	1145	6	3	2	2	2	4	58	77	1222

Class A carbape-nemase genotype	CPO class A OR unspec. carbapenemase (CARBA OR CARB) detected																	
	CARBA OR CARB absent									CARBA OR CARB present								Sum
	Meropenem MIC (CPO tested)								Sum of CARBA or CARB absent	Meropenem MIC (CPO tested)							Sum of CARBA or CARB present	
	≤ 0.125	0.25	0.5	1	2	4	8	> 8		0.25	0.5	1	2	4	8	> 8		
Neg.	583	32	24	24	48	44	16	117	888	8	10	7	23	23	16	17	104	992
Pos.					1				1	1				2	4	222	229	230
Sum	583	32	24	24	49	44	16	117	889	9	10	7	23	25	20	239	333	1222

Class B carbape-nemase genotype	CPO class B carbapenemase (CARBB) detected																	
	CARBB absent									CARBB present								Sum
	Meropenem MIC (CPO tested)								Sum of CARBB absent	Meropenem MIC (CPO tested)							Sum of CARBB present	
	≤ 0.125	0.25	0.5	1	2	4	8	> 8		0.25	0.5	1	2	4	8	> 8		
Neg.	582	37	19	24	68	66	28	298	1122	1	2	3	1	2	1		10	1132
Pos.	1		2					9	12	3	11	4	3	1	7	49	78	90
Sum	583	37	21	24	68	66	28	307	1134	4	13	7	4	3	8	49	88	1222

Class D carbape-nemase genotype	CPO class D carbapenemase (CARBD) detected																	
	CARBD absent									CARBD present								Sum
	Meropenem MIC (CPO tested)								Sum of CARBD absent	Meropenem MIC (CPO tested)							Sum of CARBD present	
	≤ 0.125	0.25	0.5	1	2	4	8	> 8		0.25	0.5	1	2	4	8	> 8		
Neg.	582	39	26	18	30	31	28	284	1038		1					1	2	1040
Pos.	1	2	3	1	2			6	15		4	12	40	38	8	65	167	182
Sum	583	41	29	19	32	31	28	290	1053		5	12	40	38	8	66	169	1222

Class D carbape-nemase genotype	CPO class D carbapenemase (CARBD) detected																	
	CARBD absent									CARBD present								Sum
	Temocillin MIC (CPO tested)								Sum of CARBD absent	Temocillin MIC (CPO tested)							Sum of CARBD present	
				≤ 4	8	16	32	> 32								> 32		
Neg.				82	228	299	188	241	1038							2	2	1040
Pos.				1				14	15							167	167	182
sum				83	228	299	188	255	1053							169	169	1222

The CPO panel was unable to classify 168 of 230 carbapenemase-encoding class A gene-positive isolates (Table 1). Yet, the CPO test flagged the presence of a carbapenemase phenotype without further classification (CARB) in 167 of these KPC-positive isolates (Table 3). Remarkably, nearly all of these merely CARB-positive strains exhibited a meropenem MIC of > 8 mg/L (Table 2). Sensitivity in classifying class A carbapenemases was 27.0%, specificity was 98.5%. A class A enzyme was indicated in the absence of a corresponding genotype in 15 of 992 isolates.

**Table 3:** Summarized data on various phenotype results of different Ambler classes tested.

Ambler class genotype present	No. of isolates	CPO detect panel phenotype				
		CARB	CARBA	CARBB	CARBD	None
**A (only)**	(228)	165	62	–	–	1
A and B	(1)	1	–	–	–	–
A and D	(1)	1	–	–	–	–
**B (only)**	(83)	5	–	75	1	2
B and D	(6)	–	–	3	3	–
D (only)	(175)	8	–	–	164	3
None	(728)	76	15	10	1	626

With respect to identifying the 90 class B gene positive isolates among the 1222 clinical isolates under investigation, the CPO detect panel demonstrated a sensitivity of 86.7% and a specificity of 99.1% (Table 1). Six class B gene positive, yet CARBB phenotype negative isolates were at least marked as CARB; one of these encoded class A and B carbapenemases (Table 3). Four class B positive and CARBB negative isolates were marked as CARBD-positive, whereby three of these contained Class B and class D encoding genes.

As regards the 182 class D gene positive isolates, assessment by the CPO detect panel resulted in a sensitivity of 91.8% and a specificity of 99.8%. All 15 isolates except one, which was not detected as CARBD among the 182 class D gene positive isolates, showed a temocillin MIC of > 32 mg/L (Table 2). Nine and three class D positive isolates were at least flagged as CARB- and CARBB-positive (Table 3).

Few isolates encoding two different Ambler classes, A and B (1), A and D (1) and B and D (6), resulted in detection of only one unspecified carbapenemase (CARB), or classification of only one of the two carbapenemase types (Table 3).

In summary, the CPO panel identified isolates as producers of any carbapenemase phenotype with an overall sensitivity of 98.8% and a specificity of 86.0%.

The sensitivity of the test in categorizing the phenotypic carbapenemase activity at the Ambler class level was 27.0% for class A, 86.7% for class B and 91.8% for class D. Nevertheless, 167 of 168 class A gene encoding strains without CARBA detection revealed a CARB positive phenotype result for unspecified carbapenemases. Only two, respectively three class B or D genotype positive isolates remained without a phenotypic result pointing to carbapenemases. Specificity in phenotypic classification was 98.5% for class A, 99.1% for class B, and 99.8% for class D.

## Discussion

This test is designed to differentiate between carbapenemase-producing organisms and resistant isolates without carbapenemases among carbapenem-resistant Gram-negative pathogens. Furthermore, fine differentiation and specification of carbapenemases into the various Ambler classes can guide antimicrobial therapy.

Previous studies on the performance of the CPO detect panel were hampered by employing strains of mainly regional origin (e.g., Louisiana, Singapore and South of Germany). Furthermore, the small number of negative control strains limited assessment of the specificity of these tests.

Here, we employed a European collection of carbapenem susceptible and non-susceptible clinical isolates from 26 European countries to evaluate the predictive capacity of the CPO detect panel.

Due to the large number of strains assessed in the study presented here, local effects and peculiarities could be largely excluded. Finally, the study is also based on a large collection of carefully collected positive as well as negative strains, which were scrutinized by WGS rather than by phenotypic microbiological comparison techniques.

All previous studies found a high sensitivity of 90–100% in the detection of carbapenemase, regardless of its classification. Our study confirmed the high sensitivity (98.8%) exhibited in detecting any carbapenemase; this is also consistent with the manufacturer's specifications of 98.3% positive agreement.

However, earlier studies found a low specificity of only 55–68%, i.e., a high rate of false positives, in contrast to the information from the manufacturer of a high negative agreement of over 95% compared to several different comparative methods. Based on a larger number of 728 characterized strains lacking carbapenemase encoding genes, we found a higher specificity of 86.0%; however, this is still limiting in practical application. Therefore, others have proposed conducting additional confirmatory rapid CPO tests to detect positive strains, such as the Carba NP test, although this is of limited practical use in daily routine. Of note, most false positive reported strains had an MIC against meropenem of 1–8 mg/L, which could lead to false conclusions regarding infection control. In 50% of cases, broad spectrum beta-lactamases and/or a loss of porins could be detected genetically (data not shown), which was also discussed in another similar study as a source of possible under-count bias^28^. Due to the large number of strains of diverse European origin local effects and peculiarities could be largely excluded.

Regarding the test performance in terms of assignment of carbapenemases to Ambler classes, an overall accuracy of over 95% is reported by the manufacturer, without further details given on the actual proportion of the three different classes evaluated. Different results have been reported by others.

Of note, assignment to class D enzymes was made with high sensitivity, although according to other technical studies a considerable proportion of overlooked carbapenemases can be assumed due to known limitations in routine diagnostics^32–34^. A limitation of this study is that it does not include all difficult-to-detect OXA carbapenemases, such as the OXA-244 carbapenemases^35^.

One study reported a sensitivity of only 69% in the classification of class B beta-lactamases^27^, mainly because 28 strains investigated were not detected. This was only partially confirmed by our study, with a more favourable result of 86.7% sensitivity in assignment of class B enzymes among 90 MBL-encoding strains tested from 10 countries.

As for class A positive strains, most of the studies including the one presented here, revealed a lower rate of correct assignments of different types of class A enzymes, in particular IMI^25^, KPC^27^, as well as KPC and GES^26^. Moreover, in our study of 230 KPC-coding strains, only 62 could be assigned to the appropriate Ambler class, although the CPO detect test indicated 167 strains to be at least positive for an unclassified carbapenemase (CARB). These isolates were from nine countries (mainly Italy with ST258/512) and included 12 multi-locus sequence typing ST (data not shown), thus excluding local peculiarities. Our data presented here offer no further explanation for this poor specificity. Clearly, this assay as delivered here does not provide a sufficient approach to detect highly resistant class A producing organisms in which the high carbapenem MIC is lowered in the presence of class A beta-lactam inhibitors, e.g., clavulanic acid.

Besides the rather limited spectrum of carbapenemases, another limitation of this carbapenemases detection study is its confinement to *K. pneumoniae*. To date, it is not known whether carbapenemase coding genes are less expressed in the genomic context of other Enterobacterales. Nevertheless, species with chromosomally expressed AmpC beta-lactamases, such as *Enterobacter cloacae* or *Klebsiella aerogenes*, which may interfere with phenotypic carbapenemase detection, were not included in the study presented here. Furthermore, glucose-nonfermenting Gram-negative bacilli (NFB), i.e. *P. aeruginosa* or *Acinetobacter baumannii* were not investigated here either, although depending on the epidemiological setting, they may account for a smaller number of CPO than in Enterobacterales.

Our genotypic categorization relies on draft genome sequencing data, which may have missed some coding sequences or lost plasmids despite the rigorous quality control of the sequences of the strains finally included (e.g., coverage, quality values of the base calls). Finally, it could be argued that for some specific resistances, DNA sequence data alone without others, such as transcriptome data, have limited predictive value^36^.

In general, demonstrating improved antimicrobial therapy decision-making based on carbapenem classification with the CPO detect panel was beyond the scope of the study presented here. This analysis did not even resistance data on relevant antibiotics, such as combinations of beta-lactamase inhibitors with carbapenems or monobactams. Further investigations are required to demonstrate a predictive value of Ambler classification in addition to antimicrobial susceptibility testing for clinical decision and outcome.

In summary, the test showed good sensitivity but lower specificity in detecting non-specific carbapenemases, with a considerable false positive rate.

This could lead to overestimation of CPO in clinical routine with all its consequences. Classification of carbapenemases employing the CPO detect panel resulted in accurate assignment to Ambler classes B and D. However, many isolates with class A enzymes were detected as unspecified CPO only, without a more accurate assignment to class A.

The overall sensitivity displayed by these tests in detecting any carbapenemase regardless of a more accurate classification was 98.8%, which appears acceptable for routine use.

However, the comparatively low specificity of 86% leads to frequent false positive detection of carbapenemase producing organisms. Previous studies were mainly based on local strain collections of predominantly resistant strains and only a small number of sensitive strains. The low specificity displayed by the CPO test (55.3–68.6%), also shown by other studies, was based in all cases on a small number of CPO-negative strains, ranging from 30 to 51.

While the test was acceptable in the detection and classification of B and D carbapenemases with a sensitivity of 90–100%, all the studies showed low sensitivity in the detection and classification of A carbapenemases with locally different predominating class A type enzymes.

This CPO detect test has two main limitations in overall specificity and classification of class A carbapenemases. The low sensitivity shown in detection of class A enzymes primarily affects the assignment to this class, but not the identification of carbapenemase activity per se.

A negative CPO detect test result is of direct practical use/predictive value, while a positive result requires further confirmatory testing.

## Methods

### Strains

Sequence data on the presence/absence of carbapenemase resistance genes and multilocus sequence type were available for a set of 1222 *K. pneumoniae* isolates from 1356 Enterobacterales based on draft genome sequence data. Isolates were recultivated overnight at 35 °C on Columbia 5% sheep blood agar from a Microbank strain collection.

### EuSCAPE AST data

All strains were extensively phenotypically characterized by the Italian National Reference Laboratory, Universita deligi Studi Firence, Dipartimento di Medicina Sperimentale e Clinica, Firence, Italy (Prof. Gianni Rossolini). In the latter investigation, a custom-made, semi-automated broth microdilution was used (Thermo Fischer Scientific, Basingstoke, Hampshire, UK). All isolates were tested against 16 antibiotic compounds of clinical relevance and 15 could be clinically categorized according to the European Committee on Antimicrobial Susceptibility Testing^37^.

### EuSCAPE sequencing data

*K. pneumoniae* isolates were genetically characterized by high-quality whole genome sequencing carried out at the Wellcome Trust Sanger Institute, Wellcome Trust Genome Campus, Hinxton, UK. All sequence data are deposited at the European Nucleotide Archive (http://www.ebi.ac.uk/ena/data/view/PRJEB10018) and have been published previously^31,38^. All reads have been mapped to relevant reference genomes and also de-novo assembled to reconstruct full draft genomes. Phylogenetic analyses of these genomes illustrate the genetic population structure of this species in Europe.

### Isolate preparation for the CPO detect test and QC

Bacterial cells were resuspended in BD Phoenix AP ID Broth and adjusted to McFarland of 0.25 by use of a BD Phoenix AP instrument (Automated Nephelometry for the BD Phoenix ID/AST System). The suspension was inoculated into the BD Phoenix NMIC-502 panel, including the CPO Detect test (in short „the CPO panel”) and placed in a BD Phoenix M50 station.

Antimicrobial testing results and carbapenemase-producer classification were interpreted using the BD EpiCenter Microbiology Data Management System (V7.20/V6.35A) with breakpoints set by EUCAST^37^.

In general, the CPO detect test provides four different mutually exclusive data outputs. Carbapenemase-phenotypes were either classified as carbapenemase-producer without further details (CARB) or phenotypically assigned to Ambler class A (CARBA), class B (CARBB) or class D (CARBD). In addition, all four carbapenemases phenotypes were grouped together as CARB_OR in this study. The electronic data were captured from the EpiCenter System and exported as a tab delimited file from the corresponding MySQL database using a script provided by BD.

Quality control was carried out on a weekly basis (*E. coli* ATCC 25922, *P. aeruginosa* ATCC 27853, *E. coli* ATCC 35218, *K. pneumoniae* ATCC 700603 or *K. pneumoniae* ATCC BAA-1705), as recommended by the manufacturer. If the QC control failed again when the isolate was retested, the isolate in question was not considered further in this study.

The MIC values determined by the EuSCAPE reference method were clinically categorized according to EUCAST^37^ and in accordance with the BD EpiCenter Microbiology Data Management System settings mentioned above. In case of discrepant categorical results between the NMIC-502 CPO detect panel and the previously determined EuSCAPE AST data of the collection, i.e., (very) major errors, the Phoenix runs were repeated, except for colistin test results. For remaining discrepancies that could not be resolved even in a repeat run, susceptibility was then determined in dilution test procedures^39^.

## References

[1] Logan LK, Weinstein RA (2017). The epidemiology of carbapenem-resistant *Enterobacteriaceae*: The impact and evolution of a global menace. J. Infect. Dis..

[2] van Duin D, Doi Y (2017). The global epidemiology of carbapenemase-producing *Enterobacteriaceae*. Virulence.

[3] Tzouvelekis LS, Markogiannakis A, Piperaki E, Souli M, Daikos GL (2014). Treating infections caused by carbapenemase-producing *Enterobacteriaceae*. Clin. Microbiol. Infect..

[4] Tsai YK (2011). *Klebsiella pneumoniae* outer membrane porins OmpK35 and OmpK36 play roles in both antimicrobial resistance and virulence. Antimicrob. Agents Chemother..

[5] Pantel A (2016). Modulation of membrane influx and efflux in *Escherichia coli* Sequence type 131 has an impact on bacterial motility, biofilm formation, and virulence in a *Caenorhabditis elegans* model. Antimicrob. Agents Chemother..

[6] Ferenci T, Phan K (2015). How porin heterogeneity and trade-offs affect the antibiotic susceptibility of gram-negative bacteria. Genes.

[7] Livermore DM, Nicolau DP, Hopkins KL, Meunier D (2020). ‘CRE, CRO, CPE and CPO’: Terminology past its ‘sell-by-date’ in an era of new antibiotics and regional carbapenemase epidemiology. Clin. Infect. Dis..

[8] Reck F (2018). Optimization of novel monobactams with activity against carbapenem-resistant *Enterobacteriaceae*—Identification of LYS228. Bioorg. Med. Chem. Lett..

[9] Felici A (1993). An overview of the kinetic parameters of class B beta-lactamases. Biochem. J..

[10] Nordmann P, Poirel L, Dortet L (2012). Rapid detection of carbapenemase-producing *Enterobacteriaceae*. Emerg. Infect. Dis..

[11] Pierce VM (2017). Modified carbapenem inactivation method for phenotypic detection of carbapenemase production among *Enterobacteriaceae*. J. Clin. Microbiol..

[12] Carvalhaes CG, Picao RC, Nicoletti AG, Xavier DE, Gales AC (2010). Cloverleaf test (modified Hodge test) for detecting carbapenemase production in *Klebsiella pneumoniae*: Be aware of false positive results. J. Antimicrob. Chemother..

[13] Anderson KF (2007). Evaluation of methods to identify the *Klebsiella pneumoniae* carbapenemase in *Enterobacteriaceae*. J. Clin. Microbiol..

[14] Tsakris A (2010). A simple phenotypic method for the differentiation of metallo-beta-lactamases and class A KPC carbapenemases in *Enterobacteriaceae* clinical isolates. J. Antimicrob. Chemother..

[15] van Dijk K (2014). A disc diffusion assay for detection of class A, B and OXA-48 carbapenemases in *Enterobacteriaceae* using phenyl boronic acid, dipicolinic acid and temocillin. Clin. Microbiol. Infect..

[16] Wareham DW, Shah R, Betts JW, Phee LM, Momin MH (2016). Evaluation of an immunochromatographic lateral flow assay (OXA-48 K-SeT) for rapid detection of OXA-48-like carbapenemases in *Enterobacteriaceae*. J. Clin. Microbiol..

[17] Glupczynski Y (2017). Prospective evaluation of the OKN K-SeT assay, a new multiplex immunochromatographic test for the rapid detection of OXA-48-like, KPC and NDM carbapenemases. J. Antimicrob. Chemother..

[18] Kaase M, Szabados F, Wassill L, Gatermann SG (2012). Detection of carbapenemases in *Enterobacteriaceae* by a commercial multiplex PCR. J. Clin. Microbiol..

[19] Monteiro J, Widen RH, Pignatari AC, Kubasek C, Silbert S (2012). Rapid detection of carbapenemase genes by multiplex real-time PCR. J. Antimicrob. Chemother..

[20] Cunningham SA, Vasoo S, Patel R (2016). Evaluation of the check-points check MDR CT103 and CT103 XL microarray kits by use of preparatory rapid cell lysis. J. Clin. Microbiol..

[21] Woodford N, Warner M, Pike R, Zhang J (2011). Evaluation of a commercial microarray to detect carbapenemase-producing *Enterobacteriaceae*. J. Antimicrob. Chemother..

[22] Koser CU (2012). Routine use of microbial whole genome sequencing in diagnostic and public health microbiology. PLoS Pathog..

[23] Pecora ND (2015). Genomically informed surveillance for carbapenem-resistant *Enterobacteriaceae* in a health care system. mBio.

[24] Mathers AJ (2015). *Klebsiella pneumoniae* carbapenemase (KPC)-producing *K. pneumoniae* at a single institution: Insights into endemicity from whole-genome sequencing. Antimicrob. Agents Chemother..

[25] Ong CH, Ratnayake L, Ang MLT, Lin RTP, Chan DSG (2018). Diagnostic accuracy of BD Phoenix CPO detect for carbapenemase production in 190 *Enterobacteriaceae* isolates. J Clin Microbiol.

[26] Simon M (2018). Evaluation of the automated BD Phoenix CPO Detect panel in combination with the beta-CARBA assay for detection and classification of carbapenemase-producing Enterobacterales. J. Microbiol. Methods.

[27] Thomson G (2017). High-stringency evaluation of the automated BD Phoenix CPO detect and Rapidec Carba NP tests for detection and classification of carbapenemases. J. Clin. Microbiol..

[28] Whitley V (2020). Multicenter evaluation of the BD Phoenix CPO detect test for detection and classification of carbapenemase-producing organisms in clinical isolates. J. Clin. Microbiol..

[29] Croxatto A (2020). Evaluation of the BD Phoenix CPO detect test for the detection of carbapenemase producers. Clin Microbiol Infect.

[30] Cho H (2020). Performance evaluation of automated BD Phoenix NMIC-500 panel for carbapenemase detection in carbapenem-resistant and carbapenem-susceptible *Enterobacterales*. J. Microbiol. Methods.

[31] David S (2019). Epidemic of carbapenem-resistant *Klebsiella pneumoniae* in Europe is driven by nosocomial spread. Nat. Microbiol..

[32] Doyle D (2012). Laboratory detection of *Enterobacteriaceae* that produce carbapenemases. J. Clin. Microbiol..

[33] Woodford N (2010). Comparison of BD Phoenix, Vitek 2, and MicroScan automated systems for detection and inference of mechanisms responsible for carbapenem resistance in *Enterobacteriaceae*. J. Clin. Microbiol..

[34] Viau R (2016). Intestinal carriage of carbapenemase-producing organisms: Current status of surveillance methods. Clin. Microbiol. Rev..

[35] Hoyos-Mallecot Y (2017). OXA-244-producing *Escherichia coli* isolates, a challenge for clinical microbiology laboratories. Antimicrob. Agents Chemother..

[36] Khaledi A (2020). Predicting antimicrobial resistance in *Pseudomonas aeruginosa* with machine learning-enabled molecular diagnostics. EMBO Mol. Med..

[37] EUCAST. EUCAST Breakpoint tables for interpretation of MICs and zone diameters Version 9.0, January 1, 2019. http://www.eucast.org/clinical_breakpoints/ (2019). http://www.eucast.org/clinical_breakpoints/.

[38] Glasner C (2013). Carbapenemase-producing *Enterobacteriaceae* in Europe: A survey among national experts from 39 countries, February 2013. Eur. Commun. Dis. Bull..

[39] CLSI (2015). Methods for Dilution Antimicrobial Susceptibility Tests for Bacteria That Grow Aerobically. Approved Standard-Tenth Edition.

